# Local immune microenvironment of skin may play an important role in the development of pretibial myxedema

**DOI:** 10.1111/exd.14402

**Published:** 2021-06-11

**Authors:** Xiaoying Chen, Jiaoyun Dong, Li Zhang, Xiaoqing Zhao, Ruofei Shi, Meng Pan, Jie Zheng

**Affiliations:** ^1^ Department of Dermatology Rui Jin Hospital Shanghai Jiao Tong University School of Medicine Shanghai China; ^2^ Department of Burn Ruijin Hospital Shanghai Jiao Tong University School of Medicine Shanghai China

**Keywords:** fibroblast, inducible skin‐associated lymphoid tissue (iSALT), pretibial myxedema (PTM), TRAb

## Abstract

Pretibial myxedema (PTM), characterized by the accumulation of glycosaminoglycans in dermis is an autoimmune skin disorder, which is almost always associated with Graves’ disease (GD). Although fibroblast stimulated by thyroid‐stimulating hormone receptor (TSHR) antibody, cytokines and growth factors have been postulated as target of the autoimmune process in the dermopathy, the pathogenesis of PTM remains unclear. We hypothesize that the local immune microenvironment of the skin including the antigens and antibodies, T cells, B cells, plasma cells and fibroblasts may play an important role in the development of PTM.

Results obtained on PTM patients indicate increased thyroid‐stimulating hormone receptor antibodies (TRAb) in the blood positively correlate with the dermal thickness of the lesions. Further analysis shows that there were more CD3+ T cells and CD20+ B cells in the skin lesions. These T and B cells are in close contact, indicating that inducible skin‐associated lymphoid tissue (iSALT) may be formed in the area. In addition, we found that the infiltrating plasma cells can secrete TRAb, proving that B cells in the skin other than the thyroid are an additional source of TSHR antibodies. Meanwhile, the T and B cells in the skin or skin homogenate of patients can promote the proliferation of pretibial fibroblasts. In conclusion, our results provide evidence that the local immune microenvironment of the skin may play an important role in the development of PTM.

## 1 BACKGROUND

Pretibial myxedema (PTM), an uncommon thyroid dermopathy, predominantly affecting patients with Graves’ disease (GD), and is characterized by brown or pink discolouration waxy appearance and bilateral lower extremity oedema.^1^ The typical histopathological features of PTM patients are the accumulation of glycosaminoglycans (GAG), mainly hyaluronic acid in reticular dermis and lymphocyte infiltration. Up to 97% of PTM cases accompanied by Graves’ ophthalmopathy (GO).^2^, ^3^ In the active phase of GO, T cells and B cells infiltrate in the orbits and activate fibroblast through IL‐17, TNFα, TGF, CD40 ligands, etc., which in turn promote the secretion of glycosaminoglycans (such as hyaluronic acid, etc.) and inflammatory molecules, finally causing tissue remodeling.^4^, ^5^, ^6^ Despite GO has been studied extensively, the mechanisms underlying the pathogenesis of PTM remain unknown. Even though infiltration of CD4+ and CD8+ T cells has been observed in PTM patients,^7^ the specific functions of these T cells or B cells have not yet systematically clarified.

## 2 PREMISES

Orbital and pretibial fibroblast has been speculated as targets of the autoimmune process in ophthalmopathy and dermopathy, supported by the presence of thyrotropin receptor (TSH‐R) immunoreactivity in the dermal and orbital fibroblasts and identification of the thyrotropin receptor antibody (TRAb)‐binding sites in the plasma membranes of fibroblasts.^8^, ^9^, ^10^

PTM shares many common features with GO. For example, both have accumulation of GAG, abnormal proliferation of fibroblasts, TSHR expression in the skin fibroblasts and orbital fibroblasts.^2^, ^11^ TSHR can activate the downstream signalling pathway by binding to TSHR antibodies,^8^ which causes the activation of fibroblast and cell proliferation, and leading to the abundant production of GAG.^12^ Additionally, the disease progression correlated with serum TRAb levels has been reported.^7^

In GD patients, B cells in thyroid are considered as the main source for TSHR antibodies. However, it is unclear that whether TRAb comes uniquely from the thyroid in PTM patients. Moreover, can other parts of the body produce TRAb to promote disease has yet to be elucidated? In addition to TRAb, T cells and B cells, the components of adaptive immunity, may also play an important role in the development of PTM. Ectopic lymphoid‐like structures (ELSs) or tertiary lymphoid organs (TLOs) are structures with an organisation similar to one of secondary lymphoid organs, including at least T cells and B cells, which can enhance antibody production.^13^ In lung inflammation or infection, induced bronchial‐associated lymphoid tissue (iBALT) is formed in the lungs where leukocyte aggregation occurs. iBALT supports initial B and T cell initiation (priming) and maintains the settlement of memory B and T cells, thereby initiating a rapid and efficient immune response in the lung during the resistance to pathogens.^14^ ELSs widely exist in the lesions of pemphigus and melanoma.^15^, ^16^ ELSs in skin lesions are regarded as a special form of inducible skin‐associated lymphoid tissue (iSALT) that generates antibodies.^17^

## 3 HYPOTHESIS

It is our hypothesis that the local immune microenvironment of the skin including the antigens and antibodies, T cells, B cells, plasma cells and fibroblasts may play an important role in the development of PTM.

## 4 HOW TO TEST THE HYPOTHESIS

40 PTM patients, four panniculitis patients and four healthy subjects were enrolled, blood samples and anterior tibial skin lesions were collected. Ultrasound was performed to record the skin thickness of the PTM patients described in our previous study.^18^ The infiltration and distribution of T cells, B cells as well as plasma cells in the lesions were examined by immunohistochemical staining and immunofluorescence staining. Plasma cell culture medium derived from the skin of PTM patients or peripheral blood of healthy subjects was harvested 4 days later; the level of TRAb in the supernatant was detected by M22‐TBII approach using an automatic electrochemiluminescence immunoassay. In addition, CD3^+^ T cells and CD38^+^/CD19^+^ B cells were collected using fluorescence‐activated cell sorting (FACS). The effects of T and B cells on the proliferation of pretibial fibroblasts were investigated by using Cell Counting Kit‐8(CCK8) kit. Details of the materials and methods are elaborated in the Supporting Information (Data S1).

The study was approved by Shanghai Jiao Tong University School of Medicine Research Ethics Committee. Written informed consent was obtained from all subjects before this study.

## 5 PRELIMINARY SUPPORTING EVIDENCE

### 5.1 Peripheral blood TRAb concentration was significantly elevated in PTM patients

**FIGURE 1:**
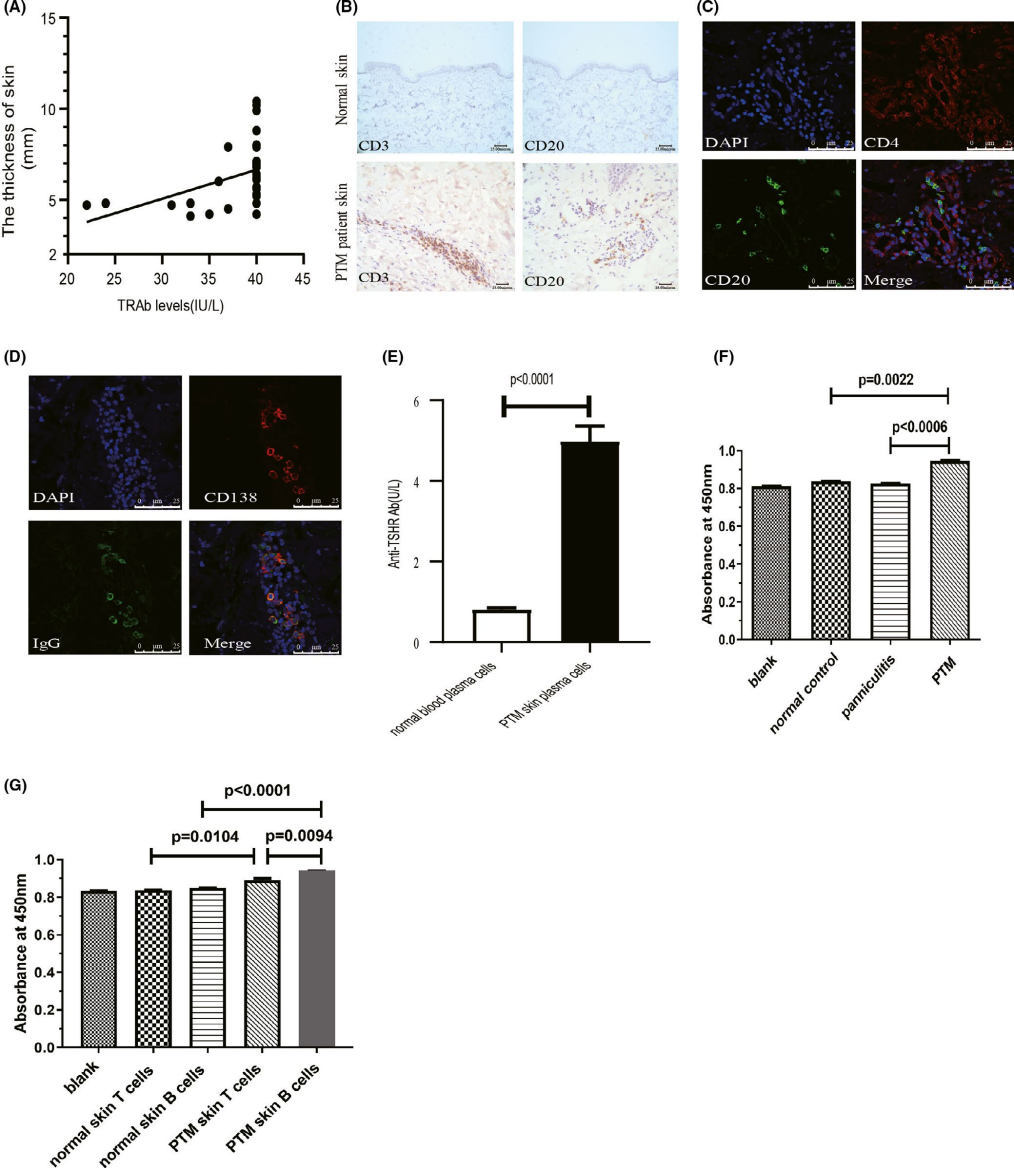
(A) Correlation of serum TRAb level and the thickness of lesions dermis (*r* = 0.538, *p* = 0.0003). (B) Immunohistochemical analysis of CD3 and CD20 expression in normal skin and PTM patients. (C) The skin sections of PTM patients were stained with anti‐CD4 antibody(red) and anti‐CD20 antibody (green) and counterstained with DAPI (blue) by immunohistochemical. (D) The skin sections of PTM patients were stained with anti‐CD138 antibody (red) and anti‐IgG antibody (green) and counterstained with DAPI (blue) by immunohistochemical. (E) Plasma cell culture medium derived from skin of PTM patients or blood of healthy subjects was harvested 4 days later; the level of TRAb was measured and summarized. (F) Effects of different skin homogenous on fibroblast proliferation. (G) Effects of T and B cells from healthy human and PTM patients’ skin on fibroblast proliferation

In our study, the titre of TRAb in all patients with PTM was significantly higher than the normal value (<1.75 U/L), 31 cases (account for 77.5%) were 40 IU/L, 7 cases (17.5%) were between 30 and 40 IU/L and 2 cases (5%) were between 20 and 30 IU/L. Meanwhile, the titter of TRAb in healthy controls is all normal. Furthermore, the titre of TRAb positively correlated with the dermis thickness of the lesion (*r* = 0.538, *p* = 0.0003) (Figure 1A).

### 5.2 T cells and B cells infiltrated in PTM lesions

Having observed the infiltration of lymphocyte in the dermis of PTM patients by HE staining, we then examined the distribution of T and B cells by immunohistochemistry. We found that there were no significant infiltrating T cells or B cells in normal pretibial skin, whereas amounts of CD3^+^ T cells and a small population of CD20^+^ mature B cells assembled in pretibial in approximately 77.5% (31cases) of PTM patients (Figure 1B). Moreover, we noticed that those infiltrating CD20^+^ B cells were distributed and closely attached with the CD4^+^ T cells by immunofluorescence staining (Figure 1C). Besides the mature B cells, CD138^+^ plasma cells which can secrete antibodies are also present in the skin of PTM patients. Remarkably, IgG signal can be detected on the surface of these CD138^+^ plasma cells, suggesting that these plasma cells are secreting antibodies at local lesions (Figure 1D).

### 5.3 Plasma cells infiltrated in PTM lesions can produce TRAB

As described above, the plasma cells infiltrated into PTM lesions can produce IgG antibodies, we investigated whether PTM associated antibodies, such as TRAb, were retained. The plasma cells dissociated from normal blood and the skin lesions of PTM patients were cultured in vitro. After being cultured for four days, cell culture medium was collected to examine the concentration of TRAb by M22‐TBII approach. Markedly increased levels of TRAb were detected in PTM patients’ culture medium, compared to the normal blood culture medium (Figure 1E). These data indicated that these plasma cells infiltrated in the lesions have the ability to secrete TRAb.

### 5.4 PTM patient‐derived T cells and B cells promoted fibroblast proliferation

It has been reported that TSH‐R is present on the surface of skin fibroblasts, and the immunoglobulins from the serum of GD patients can directly promote fibroblast proliferation through TSH‐R.^8^ We then studied whether those immune globulin or cytokines secreted by the infiltrating T and B cells in the PTM patients directly stimulate fibroblasts proliferation. To address this question, we sought to examine the proliferation of fibroblasts after 1‐day treatment with skin homogenate from healthy controls, panniculitis patients as well as PTM patients, respectively. We found that only the homogenate from the skin of PTM patients was able to promote fibroblasts proliferation, while the other homogenates had no effects, suggesting that the skin of PTM patients has localized antibodies or cytokines that promote fibroblast proliferation (Figure 1F). To further investigate whether such promoting effect was due to infiltrating T and B cells in the skin of PTM patients, we isolated T and B cells from PTM patients and healthy human skin, and co‐cultured them with fibroblasts, respectively. At 72 h after co‐culture, fibroblasts proliferation was examined by CCK‐8 assay. The proliferation of fibroblasts was enhanced in the presence of T cells or B cells isolated from PTM patients, while no alterations in fibroblast cell proliferation were found after co‐culture with those T cells or B cells originated from normal human skin (Figure 1G). Our findings suggested that localized infiltration of T and B cells in the skin of PTM patients may directly promote the progression of PTM disease via stimulating fibroblast proliferation and activation.

## 6 RELEVANCE AND PERSPECTIVES

This study is the first to explore that T and B cells clustered together to form iSALT like structures in the lesions of PTM, where T and B cells are in close contact with each other, and CD138^+^ plasma cells that secrete IgG also settled. In summary, our study identified the presence of iSALT‐like structure in the lesions of PTM patients, and T or B cells in this structure were able to promote the proliferation of fibroblasts, suggesting that the immune microenvironment of the skin may play critical role in PTM development.

## CONFLICT OF INTEREST

The authors declare no conflict of interest.

## AUTHOR CONTRIBUTIONS

XC and JD designed the research study and wrote the paper; MP performed the statistical analysis and manuscript revision; RS collected the blood and skin samples; LZ and XZ performed the experiments; JZ revized the paper. All authors have read and approved the final manuscript.

## Supporting information


**Fig S1.** Supplementary figure

**Supplementary Material** Materials and methods

## Data Availability

The data that support the findings of this study are available from the corresponding author upon reasonable request.
