# Supplementary figures and images for: Local immune microenvironment of skin may play an important role in the development of pretibial myxedema
Source: Exp Dermatol. 2021 Jun 11;30(12):1820–4. doi: 10.1111/exd.14402 (PMC8597019; doi:10.1111/exd.14402)

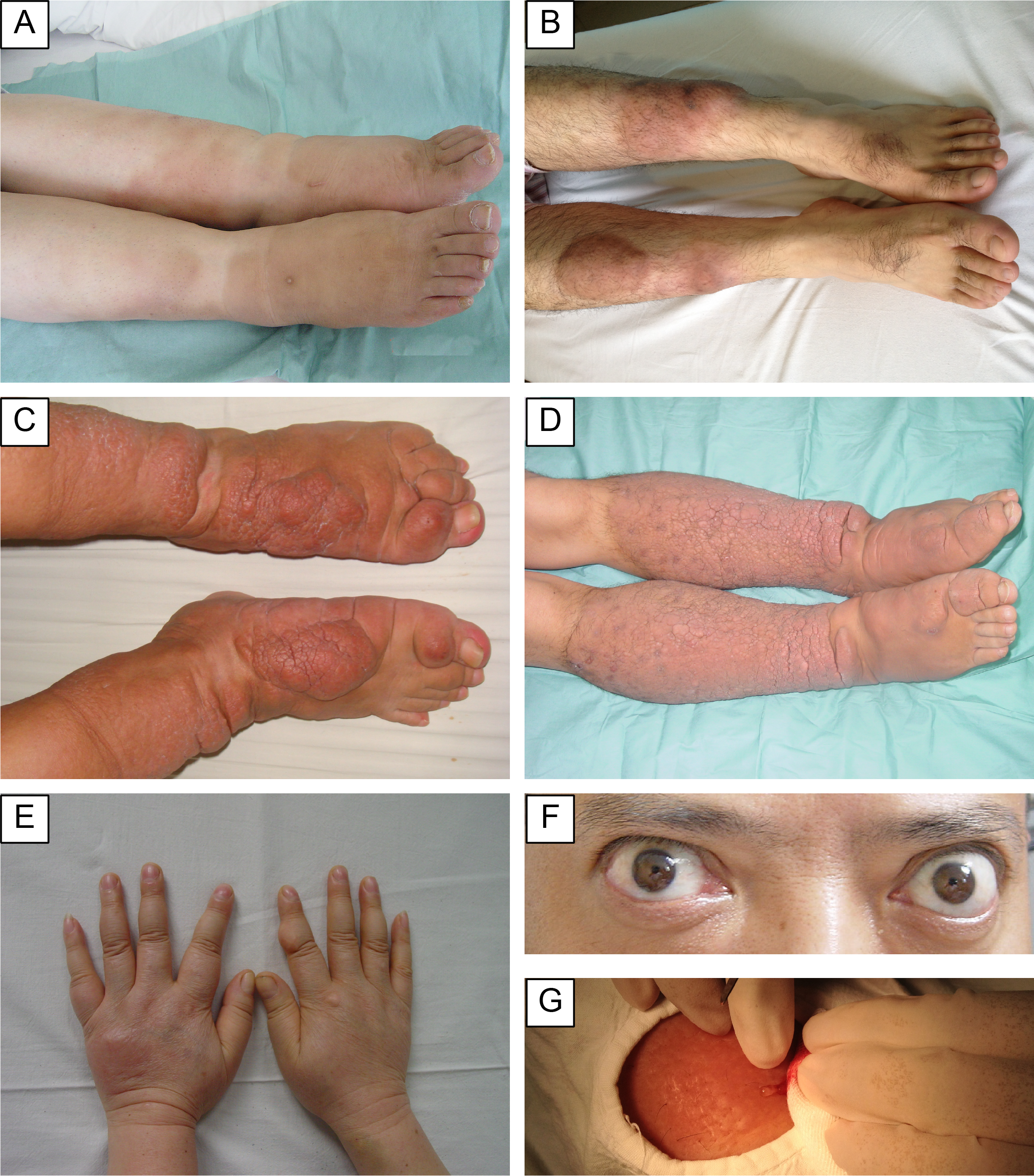

Supplement: Supplementary file 1 — Fig S1. Supplementary figure [file EXD-30-1820-s001.png]
