# Supplementary material for: Local immune microenvironment of skin may play an important role in the development of pretibial myxedema
Source: Exp Dermatol. 2021 Jun 11;30(12):1820–4. doi: 10.1111/exd.14402 (PMC8597019; doi:10.1111/exd.14402)
Supplement: Supplementary file 2 — Supplementary Material Materials and methods [file EXD-30-1820-s002.docx]

**1. Clinical Characteristics of PTM Patients**

A total of 40 PTM patients enrolled in our department from January 2015 to December 2018 were recruited in our study. The age of these subjects ranges from 23 to 67 years old, including 24 males and 16 females, at average age of 44.6 ± 13.0 years old; The mean duration of PTM and GD was 2.9 ± 2.7 years (range from 0.5 month to 9 years), 5.1 ± 4.8 years (range from 3 month to 20 years), respectively.

Within these PTM patients, we identified four major distinct type: (1) edema form (15/40) that the skin exhibits diffuse non-pitting edema with the slightly thicken dermis, edema may spread to 1/4 to 1/3 length of the legs (Figure S1A); (2) plaque form(11/40), in which >1cm red or dark plaques can be seen on the surface of the skin, sometimes accompany by localized hypertrichosis or [orange](https://www.sciencedirect.com/topics/medicine-and-dentistry/peau-dorange)-like appearance (Figure S1B); (3) nodular form(9/40) that mono or multiple clear circumscribed nodules were observed and easily touched by hands, which may be elevated or not (Figure S1C); (4) elephantiasis form(5/40), which exhibited signs of significantly diffuse edema, plaques and nodules or lumps, and dispersed in a smooth or polyps-like surface of the skin (Figure S1D). In addition, 7 PTM patients were accompanied by thyroid acropachy, which showed enlarged fingers (Figure S1E). We also observed that 38/40 PTM patients have Graves' ophthalmopathy, signs of bulging eyes and retracted eyelids (Figure S1F). Besides, abundant of clear gelatinous mucus accumulated under the lesion in two non-pitting edema patients was observed in the skin biopsy (Figure S1G).

**2. Ultrasound measurement of dermal thickness**

Ultrasound was performed to record skin thickness. Specifically, measurements were made at the thickest site in PTM patients with the plaque and nodular type or at the lower 1/4 of the distance from lateral malleolus to head of fibular in edematous and elephantiasic type.

**3. Thyroid hormone receptor antibody (TRAb) assay**

The titre of TRAb in the patients’ serum was detected by M22-TBII method using an automatic electrochemiluminescence immunoassay. The normal TRAb level in serum is 0-1.75 IU/L. While written as 40 IU/L if the value is greater than 40 IU/L.

**4. Histopathology analyses**

A portion of the skin sample was fixed by 4% paraformaldehyde (PFA), dehydrated with serial ethanol and embedded in paraffin, which were used for [hematoxylin](https://www.sciencedirect.com/topics/medicine-and-dentistry/haematoxylin) and [eosin](https://www.sciencedirect.com/topics/medicine-and-dentistry/eosin)(H&E) staining, Alcian blue staining and immunohistochemical staining. For immunohistochemical staining, sections (3-5 μm) were stained for anti-human CD3 (clone: UCHT1,1:200) or anti-human CD20 (clone: L26,1:200) overnight at 4°C after deparaffinize, rehydrate, and antigen retrieval in sodium citrate (PH 6.0). ABC Reagents (Vector Laboratories, PK-4001) and ImmPACT DAB substrate (Vector Laboratories, SK-4105) were prepared and used according to standard protocol. For immunofluorescence staining, the sections were blocked for 1hr with normal goat serum at room temperature (RT), stained with primary antibodies overnight at 4°C, followed by incubation with secondary antibodies for 3hr at RT in light-protected box. The following primary antibodies or IgG control were used at 1:200 dilution, including anti-human CD4 (clone: RPA-T4), anti-human CD20 (clone: L26), anti-human CD138 (clone: MI15), anti-human CD138 (clone: EPR4421). Samples were counterstained with DAPI and mounted. All images were observed and captured using an Olympus BX53 microscope or the Lecia TCS SP8 microscope.

**5. Fibroblast culture and cell proliferation assay**

Fibroblasts were obtained from the normal human tibial anterior skin by using tissue culture approach. Isolated fibroblast cells were then cultured in F10 medium supplemented with 10% fetal calf serum, 1% glutamine, and 1% penicillin/streptomycin. Fibroblasts were plated at a density of 2 x 10^3^ cells/well in 96-well plate. After being cultured 24hrs, cells were treated with 100ul skin homogenate obtained from healthy human, panniculitis patient or PTM patient, respectively. After treatment for 24hrs, replace the skin homogenate medium with fresh culture medium. The cell proliferation was detected using Cell Counting Kit-8(CCK8) kit. The skin homogenate was centrifuged at 10,000 rpm for 10 minutes to collect the anterior tibial skin culture supernatant.

For co-culture experiments, skin samples were digested in collagenase I-A (80 mg/ml) and DNase I (5 MU/ml) solution for 60 minutes at 37 °C, then triturated to obtain the single cell suspension assisting with syringe. Obtained single cells were filtered (70um), then immune cellswere enriched from the suspension by CD45 magnetic beads. CD3^+^ T cells and CD38^+^/CD19^+^ B cells were then collected using fluorescence-activated cell sorting (FACS). Normal human T and B cells from blood also can be harvested by the same method. The purified T cells or B cells (5 x 10^3^ cells/well) were then added in the fibroblast pre-seed 96-wells, and co-culture for 3 days. After that, removing the suspended cells, and utilize the CCK8 kit to detect the proliferation capacity of attached fibroblasts.

**6. Plasma cell culture and medium TRAb assay**

Blood from healthy controls and skin tissue from PTM patients were dissociated into the single-cell suspension, then were incubated with an FITC-conjugated antibody against CD138 (clone: MI15) for 20 minutes at 4°C. FITC positive selection kit (Stem cell) was used to bind the FITC antibody-labeled cells. The plasma cells originated from healthy human blood or PTM patients’ skin lesions were further purified using FACs. Cells were seeded at a density of 2 x 10^3^ cells/well in 96-well plates and maintained in F10 medium containing 10% FCS and 10 ng/ml human r-IL-6 (R&D). After being cultured for 4 days, the supernatant was harvested after centrifugation. As described above, the level of TRAb in the supernatant was detected by M22-TBII approach using an automatic electrochemiluminescence immunoassay.

**7. Statistical Analysis**

Data handling and statistical processing was conducted using GraphPad Prism Software. Unless mentioned otherwise, all data were expressed as mean value ± standard deviation (SD). Unpaired Student’s *t* test (two-tailed) was performed to analyze the statistically significant difference in patients and controls. *p< 0.05* was considered significantly difference. The statistical relationship between variables was analyzed using the Spearman's correlation.
